# Determinants of Exposure to Second-Hand Tobacco Smoke (SHS) among Current Non-Smoking In-School Adolescents (aged 11–18 years) in South Africa: Results from the 2008 GYTS Study

**DOI:** 10.3390/ijerph8093553

**Published:** 2011-08-31

**Authors:** Karl Peltzer

**Affiliations:** 1 HIV/AIDS/STI/and TB (HAST), Human Sciences Research Council, 134 Pretorius Street, Pretoria 0002, South Africa; E-Mail: Kpeltzer@hsrc.ac.za; Tel.: +27-12-302-2000; Fax: +27-12-302-2601; 2 Department of Psychology, University of Limpopo, P/B X1106, Sovenga 0727, South Africa

**Keywords:** environmental tobacco smoke, attitudes, school going adolescents, South Africa

## Abstract

The aim of this study was to estimate the prevalence and identify correlates of second-hand tobacco smoke (SHS) among 6,412 current non-smoking school-going adolescents (aged 11 to 18 years) in South Africa. A cross-sectional study was carried out in 2008 in South Africa within the framework of the Global Youth Tobacco Survey. Overall, 25.7% of students were exposed to SHS at home, 34.2% outside of the home and 18.3% were exposed to SHS at home and outside of the home. Parental and close friends smoking status, allowing someone to smoke around you and perception that passive smoking was harmful were significant determinants of adolescent’s exposure to both SHS at home and outside of the home. Identified factors can inform the implementation of public health interventions in order to reduce passive smoking among adolescents.

## 1. Introduction

Worldwide, 40% of children, 33% of male non-smokers, and 35% of female non-smokers, were exposed to Second-hand Tobacco Smoke (SHS) in 2004, while in Africa 12.5% of children were exposed to SHS [1]. Among school-going adolescents in Uganda 17.9% were exposed to SHS at home while 48.7% were exposed to SHS outside of the home [2]. Exposure to SHS in children contributes significantly to morbidity and mortality [1,3]. Gender, age group, parental and close friends smoking status [2,4–6], and low perceived susceptibility [7] were found to be significantly associated with adolescent’s exposure to SHS.

The Global Youth Tobacco Survey (GYTS) is a school-based tobacco specific survey that focuses on adolescents aged 13–15 years. It assesses students’ attitudes, knowledge and behaviours related to tobacco use, and second-hand tobacco smoke exposure [8]. Prevalence estimates of SHS in a number of countries among the GYTS study participants have been reported [1]. However, the author is unaware of any studies that have studied the determinants of SHS among adolescents in South Africa. Therefore, this study aims to report the prevalence of SHS exposure and determinants of exposure among school-going adolescents in South Africa.

## 2. Methods

### 2.1. Sample and Procedure

This study involved the secondary analysis of the Global Youth Tobacco Survey (GYTS) conducted in South Africa among students in Grades 8 through 10 conducted in 2008. A two-stage cluster sample design was used to produce representative data for South Africa. At the first stage, schools were selected with probability proportional to enrolment size. At the second stage, classes were randomly selected and all students in selected classes were eligible to participate. Self-completed questionnaires were used. The school response rate was 94.6%, the class response rate was 100.0%, the student response rate was 82.4% and the overall response rate was 77.9%. All students in selected classes attending school on the day of the survey were eligible to participate [9]. Further sampling details [9]. Main characteristics between the respondents and non-respondents differed in terms of boys (P < 0.000) and lower school Grade (P < 0.000).

### 2.2. Measure and Data Analysis

The GYTS questionnaire included data on demographic variables and experience with cigarette smoking. Responses to the following questions were used in this analysis: Do you smoke? Do your parents smoke? Do any of your closest friends smoke cigarettes? Are you in favour of banning smoking in public places (such as in restaurants, in buses and trains, in schools, on playgrounds, in gyms and sports arenas, in discos/clubs)? Do you think a person who smokes around others should ask for their permission to smoke? If someone asks permission to smoke around you do you let them? Do you think the smoke from other people’s cigarettes is harmful to you? During the past 7 days, on how many days have people smoked in your home, in your presence? During the past 7 days, on how many days have people smoked in your presence, in places other than in your home? SHS exposure was defined as having had people smoke in one’s presence on one day or more in the last 7 days. Current non-smokers were defined as having smoked on zero days in the past month using the question “During the past 30 days (one month), on how many days did you smoke cigarettes? Response options were 0, 1 or 2, 3 to 5, 6 to 9, 10 to 19, 20 to 29, to all 30 days.”

A weighting factor was used in the analysis to reflect the likelihood of sampling each student and to reduce bias by compensating for differing patterns of non response. The weight used for estimation is given by the following formula: W = W1 × W2 × f1 × f2 × f3 × f4 where W1 = the inverse of the probability of selecting the school; W2 = the inverse of the probability of selecting the classroom within the school; fl = a school-level non response adjustment factor calculated by school size category (small, medium, large); f2 = a class-level non response adjustment factor calculated for each school; f3 = a student-level non response adjustment factor calculated by class; and f4 = a post stratification adjustment factor calculated by grade [8].

Data analysis was performed using STATA software version 10.0 (Stata Corporation, College Station, Texas, USA). To account for the complex sampling design and to obtain accurate variance estimates, the *svy* estimation commands for complex survey data in Stata was used to complete all analyses. Analyses conducted include the prevalence of SHS and association between SHS exposure and age, gender, smoking status of parents and closest friends and passive smoking attitudes. Unconditional logistic regression analysis was conducted to estimate associations between relevant predictor variables and SHS. Unadjusted Odds Ratios (OR) for selected predictor variables are reported while considering exposure to second-hand tobacco smoke at home and outside the home separately as dependent variable. Thereafter the results of adjusted odds ratios (AOR) for the factors are reported. All statistical inferences were based on a significance level of P ≤ 0.05 (two-sided).

## 3. Results

### 3.1. Sample Characteristics

The sample included 6,412 current non-smokers, of whom 55.4% were female in-school adolescents. The prevalence of current non-smokers in the total sample was 83.5%, 16.5% were current smokers. Overall, 25.7% of participants were exposed to SHS at home, 34.2% outside of the home, and 18.3% were exposed to SHS at home and outside of the home. Two in five (40.7%) did not think that the smoke from other people’s cigarettes was harmful to them, and just over half (53.9%) were in favour of banning smoking in public places (see Table 1).

### 3.2. Associations to Exposure to Second-Hand Tobacco Smoke (SHS)

In univariate analysis parental or guardian smoking, peer smoking, asking for permission to smoke around you, allowing someone to smoke around you and perception that passive smoking is harmful was associated with exposure to SHS at home and SHS at home and outside the home, while in multivariate analysis parental or guardian smoking, peer smoking, allowing someone to smoke around you and perception that passive smoking is harmful were retained as associated with exposure to SHS at home and at home and outside the home. In univariate analysis parental or guardian smoking, peer smoking, asking for permission to smoke around you, allowing someone to smoke around you, in favour of banning smoking in public places and perception that passive smoking is harmful was associated with exposure to SHS outside the home, while in multivariate analysis all factors were retained in the analysis (see Tables 2 and 3).

## 4. Discussion

The study found a moderate exposure to SHS among this sample of school-going current non-smoking adolescents in South Africa in 2008**,** 25.7% were at home and 34.3% were outside of the home exposed to SHS. There seems to be a reduction of exposure to SHS at home among current non-smoking school-going adolescents using GYTS results from 32.1% in 1999, to 26.2% in 2002 and 25.7% in 2008 and likewise a reduction of exposure to SHS outside the home from 41.2% in 1999, to 32.4% in 2002 but an increase to 34.2% SHS exposure outside the home [10,11]. A reduction of exposure to SHS to 2002 can be explained by the introduction of the tobacco control act in 2000 which limited smoking in public places, and a reduction of exposure to SHS at home can be attributed to an overall reduction of smoking in the South African population [12].

The study found in multivariate analysis that parental, close friends smoking status, allowing someone to smoke around you and perception that passive smoking is harmful were significant determinants of adolescent’s exposure to both SHS at home and outside of the home. Regarding SHS exposure at home the effect of parental smoking than that of close friends smoking status, which was also found in other studies [6]. Maternal/female guardian smoking had the highest impact on SHS exposure among the students studied. This finding is confirmed in a study among children in Cape Town [13]. Contrary to the study by Li *et al.* [7] where perceived susceptibility was associated with adolescent’s exposure to SHS, this study found that students with perceptions of higher degrees of harmfulness of passive smoking were more exposed to SHS. One would have expected that higher awareness of the harmfulness of passive smoking would have led to reduced exposure to SHS. Due to the students having a long-term SHS exposure at home, some of them may already have adverse effects (e.g., cough, sputum, *etc.*); they got some knowledge about harmful effects of SHS from other sources. However, they have to continue to be exposed to SHS (especially at home) because they do not have choices. The study did not find any gender and age group differences regarding exposure to SHS, which was also found in a study among Puerto Rican children [14], but other studies found higher exposure to SHS with increasing age and among males [4,6].

Study limitations include the self-reported information collection where bias may occur and that the study sample was school-based and therefore not entirely representative of all adolescents in South Africa. In addition, biomarkers [15] were not used in order to assess the SHS exposure status of the participants. However, in a study among primary school children in Turkey urinary cotinine measurements of children were highly consistent with the self-reported exposure levels (P < 0.001) [16].

## 5. Conclusions

The study found a moderate prevalence of exposure to SHS (at home and outside the home) among a national sample of school-going adolescents in South Africa. Parental, close friends smoking status, tolerance and high perceived harm of passive smoking were significant determinants of adolescent’s exposure to SHS. Public health interventions to reduce SHS exposure among adolescents may target parental or guardian in particular maternal or female guardian smoking and emphasis on the harmful effects of passive smoking in life skills education. Preventing exposure to cigarette smoke in childhood has significant potential to improve children’s health worldwide [17].

## Figures and Tables

**Table 1:** Selected sample characteristics of South African non-smoker in-school adolescents aged 11 to 18 years (2008).

	Males N (%) [95% CI]	Females N (%) [95% CI]	Total N (%) [95% CI]

**Gender**	2,794 (44.6) [41.9–47.3]	3,618 (55.4) [52.7–58.1]	6,412 (100)

**Age**			
14 or younger	612 (21.2) [17.7–24.8]	958 (25.7) [21.1–30.3]	1,585 (23.7) [20.0–27.4]
15	642 (23.1) [18.8–27.5]	957 (27.9) [23.7–32.1]	1,605 (25.7) [21.6–29.7]
16	612 (22.5) [19.3–25.7]	815 (22.6) [19.8–25.3]	1,437 (22.6) [20.0–25.1]
17	457 (16.5) [13.8–19.3]	472 (13.8) [10.4–17.3]	936 (15.1) [12.4–17.8]
18 or older	466 (16.6) [13.2–19.9]	406 (10.0) [7.9–12.1]	880 (13.0) [10.5–15.4]

**Parents or guardians smoke**			
None	1,750 (70.4) [66.9–74.0]	2,173 (68.4) [65.4–71.5]	3,962 (69.5) [66.6–72.4]
Both parents or guardians	185 (5.7) [4.2–7.2]	263 (6.3) [5.0–7.5]	453 (6.0) [4.8–7.2]
Father or male guardian only	503 (19.5) [17.0–22.0]	708 (20.6) [18.3–22.9]	1.215 (20.0) [18.1–21.8]
Mother or female guardian only	145 (4.4) [3.3–5.4]	202 (4.7) [3.5–6.0]	348 (4.5) [3.7–5.4]

**Friends smoking**			
None	1,690 (61.9) [58.7–65.1]	2,739 (77.2) [74.7–79.7]	4,429 (70.4) [68.3–72.6]
Some	857 (30.4) [27.3–33.4]	655 (17.9) [15.4–20.4]	1,512 (23.4) [21.4–25.4]
Most or All	215 (7.7) [6.3–9.1]	190 (4.9) [3.9–5.8]	405 (6.2) [5.2–7.1]

**In favour of banning smoking in public places**	1,406 (52.0) [48.1–55.9]	1,948 (55.5) [51.6–59.4]	3,354 (53.9) [50.4–57.5]

**Do you think a person who smokes around others should ask for their permission to smoke?**	1,769 (64.3) [60.7–67.9]	2,417 (67.6) [63.1–72.2]	4,186 (65.9) [62.0–69.8]

**If someone asks permission to smoke around you, do you let them?**			
Never	1,749 (63.5) [61.0–66.1]	2,574 (73.5) [70.8–76.1]	4,323 (68.9) [67.0–70.8]
Sometimes	572 (19.9) [17.4–22.5]	634 (16.7) [14.1–19.2]	1,206 (18.1) [16.3–19.9]
Always	434 (16.5) [14.7–18.4]	369 (9.9) [8.6–11.1]	803 (13.0) [11.8–14.2]

**Do you think the smoke from other people’s cigarettes is harmful to you?**			
Definitely not	1,034 (38.0) [35.1–40.9]	1,065 (29.8) [27.2–32.4]	2,099 (33.5) [31.2–35.4]
Probably not	227 (7.8) [6.3–9.3]	263 (6.7) [5.4–8.0]	490 (7.2) [6.1–8.3]
Probably yes	335 (11.8) [10.2–13.5]	447 (13.1) [11.5–14.7]	782 (12.6) [11.3–13.8]
Definitely yes	1,161 (42.3)[38.1–46.5]	1,796 (50.4)[45.8–55.1]	2,957 (46.6) [42.3–50.9]

**During the past 7 days, on how many days have people smoked in your home, in your presence**	720 (25.0) [22.2–27.8]	999 (26.3) [23.4–29.2]	1,719 (25.7) [23.0–28.3]

**During the past 7 days, on how many days have people smoked in your presence, in places other than in your home?**	977 (34.6) [31.5–37.7]	1,283 (34.1) [31.3–36.9]	2,260 (34.2) [31.6–36.8]

**Second-hand tobacco smoke at home and outside the home**	522 (17.8) [15.7–19.9]	712 (18.9) [16.4–21.3]	1,234 (18.3) [16.3–20.3]

**Table 2:** Variables associated with exposure to Second-hand Tobacco Smoke (SHS) at home or outside the home among South African current non-smoker in-school adolescents aged 11 to 18 years.

Variable	Home		Outside of the home	

	OR (95% CI)^1^	AOR (95% CI)^2^,^3^	OR (95% CI)^1^	AOR (95% CI)^2^,^4^

**Gender**				
Male	1.00		1.00	
Female	0.93 (0.84–1.05)		0.93 (0.84–1.05)	

**Age**				
14 or younger	1.00		1.00	
15	0.97 (0.75–1.26)		1.10 (0.85–1.41)	
16	0.97 (0.77–1.21)		1.14 (0.89–1.44)	
17	0.87 (0.68–1.11)		0.83 (0.61–1.14)	
18 or older	0.96 (0.71–1.29)		1.04 (0.80–1.36)	

**Parents or guardians smoke**				
None	1.00	1.00	1.00	1.00
Both parents or guardians	5.76 (4.12–8.04)***	5.45 (2.67–8.10)***	2.25 (1.77–2.86)***	2.28 (1.67–3.11)***
Father or male guardian only	4.34 (3.58–5.37)***	4.25 (3.41–5.30)***	2.10 (1.83–2.41)***	1.87 (1.58–2.20)***
Mother or female guardian only	6.36 (4.20–9.74)***	6.62 (4.09–10.71)***	3.00 (2.02–4.45)***	2.85 (1.85–4.38)***

**Friends smoking**				
None	1.00	1.00	1.00	1.00
Some	2.30 (1.91–2.76)***	2.13 (1.72–2.63)***	2.22 (1.87–2.62)***	2.20 (1.87–2.59)***
Most or All	2.31 (1.77–3.02)***	2.12 (1.52–2.96)***	2.06 (1.62–2.62)***	2.05 (1.60–2.63)***

In favour of banning smoking in public places	1.23 (0.99–1.54)		1.64 (1.34–2.01)***	1.23 (1.00–1.51)*

Do you think a person who smokes around others should ask for their permission to smoke?	1.52 (1.36–1.70)***	1.11 (0.95–1.30)	1.84 (1.57–2.15)***	1.21 (1.03–1.41)*

If someone asks permission to smoke around you, do you let them? (Sometimes or Always)	1.52 (1.36–1.70)***	1.45 (1.30–1.62)***	1.21 (1.10–1.35)***	1.16 (1.05–1.26)**

Do you think the smoke from other people’s cigarettes is harmful to you?				
Definitely not	1.00	1.00	1.00	1.00
Probably not	1.74 (1.27–2.38)***	1.38 (0.98–1.95)	2.09 (1.51–2.90)***	2.10 (1.44–3.08)***
Probably yes	2.45 (1.87–3.22)***	1.96 (1.39–2.75)***	3.43 (2.49–4.72)***	2.98 (2.17–4.11)***
Definitely yes	2.00 (1.53–2.61)***	2.01 (1.57–2.60)***	2.87 (2.22–3.71)***	2.65 (2.11–3.33)***

* P < 0.5;

** P < 0.01;

*** P < 0.001;

1 Unadjusted Odds Ratios with 95% Confidence Interval;

2 Adjusted Odds Ratios with 95% Confidence Interval;

3 Hosmer and Lemeshow Chi-square 8.34, p.30; Cox & Snell R^2^ 0.15; Nagelkerke R^2^ 0.22;

4 Hosmer and Lemeshow Chi-square 7.72, p.36; Cox & Snell R^2^ 0.09; Nagelkerke R^2^ 0.12.

**Table 3:** Variables associated with exposure to Second-hand Tobacco Smoke (SHS) at home and outside the home among South African current non-smoker in-school adolescents aged 11 to 18 years.

Variable	Second-hand tobacco smoke at home and outside the home	
	OR (95% CI)^1^	AOR (95% CI)^2^,^3^

**Gender**		
Male	1.00	
Female	0.93 (0.79–1.09)	

**Age**		
14 or younger	1.00	
15	1.15 (0.85–1.56)	
16	1.13 (0.87–1.45)	
17	0.94 (0.69–1.26)	
18 or older	1.26 (0.95–1.68)	

**Parents or guardians smoke**		
None	1.00	1.00
Both parents or guardians	4.60 (3.32–6.36)***	4.40 (3.03–6.40)***
Father or male guardian only	3.88 (3.14–4.79)***	3.59 (2.88–4.47)***
Mother or female guardian only	5.79 (3.78–8.86)***	5.73 (3.56–9.21)***

**Friends smoking**		
None	1.00	1.00
Some	2.53 (2.13–3.00)***	2.36 (1.99–2.81)***
Most or All	2.57 (1.94–3.40)***	2.31 (1.67–3.20)***

In favour of banning smoking in public places	1.23 (0.96–1.57)	

Do you think a person who smokes around others should ask for their permission to smoke?	1.51 (1.30–1.77)***	1.01 (0.84–1.21)

If someone asks permission to smoke around you, do you let them? (Sometimes or Always)	1.45 (1.30–1.62)***	1.31 (1.14–1.49)***

Do you think the smoke from other people’s cigarettes is harmful to you?		
Definitely not	1.00	1.00
Probably not	1.56 (1.05–2.31)*	1.29 (0.89–1.88)
Probably yes	2.61 (2.02–3.37)***	2.20 (1.60–3.02)***
Definitely yes	2.11 (1.66–2.68)***	2.12 (1.66–2.71)***

* P < 0.5;

** P < 0.01;

*** P < 0.001;

1 Unadjusted Odds Ratios with 95% Confidence Interval;

2 Adjusted Odds Ratios with 95% Confidence Interval;

3 Hosmer and Lemeshow Chi-square 8753.75, p.000; Cox&Snell R^2^ 0.12; Nagelkerke R^2^ 0.19.
